# A risk prediction model for recurrence in patients with borderline ovarian tumor based on artificial neural network: development and validation study

**DOI:** 10.1186/s13048-025-01920-y

**Published:** 2025-12-10

**Authors:** Qiulin Ye, Yue Qi, Chi Fei, Juanjuan Liu, Yuexin Hu, Dongying Wang, Xiao Li, Bei Lin, Tianmin Xu, Aimin Liu

**Affiliations:** 1 Department of Obstetrics and Gynecology, Shengjing Hospital of China Medical University, No.36 Sanhao Street, Shenyang, Liaoning 110004 People’s Republic of China; 2 Department of Obstetrics and Gynaecology, The Second Hospital of Jilin University, 218 Zi Qiang Street, Changchun, Jilin 130041 People’s Republic of China

**Keywords:** Borderline ovarian tumor, Recurrence prediction, Artificial neural network, Neural multi-task logistic regression

## Abstract

**Background:**

Effective management of borderline ovarian tumors (BOTs) requires timely identification of patients at high risk of recurrence. Previous studies suggest that artificial neural networks can improve the prediction of BOT recurrence compared to traditional models, though concerns about their validity persist due to insufficient external validation. We aimed to evaluate the predictive performance of a time-dependent artificial neural network, conduct comprehensive temporal and spatial external validations to address this critical limitation.

**Methods:**

Clinical data were collected from patients diagnosed with BOT at Shengjing Hospital of China Medical University between January 2014 and August 2023, including 76 cases of recurrence and 584 cases of non-recurrence. Using the Synthetic Minority Oversampling Technique (SMOTE), we balanced the groups at a 1:1 ratio (total sample size, *N* = 1168). Random sampling was used to divide the data into a training set (70%) and an internal validation set (30%). Temporal (same center; May 2011–December 2013) and spatial (different center; April 2011-April 2019) external validation sets were established. The training set data were input into the input layer of the neural network, high-level features were extracted via neurons in the hidden layer, and the model output was generated from the output layer. The model’s sensitivity, specificity, positive predictive value (PPV), negative predictive value (NPV), accuracy, time-dependent area under the receiver operating characteristic curve (tdAUC), and integrated Brier score (IBS) were evaluated using the internal validation set, the temporal external validation set, and the spatial external validation set, respectively.

**Results:**

A neural multi-task logistic regression model (N-MTLR) was constructed based on 34 features from the training set after correlation screening, with 9 variables selected for the neural network prediction model. The prediction model consisted of three functional layers with 128, 64, and 32 neurons, respectively, totaling 224 neurons. The optimal parameters for the final model were set as follows: initialization method of glorot_uniform, Dropout rate of 30%, L2 regularization parameter of 1e-2, optimizer of Adam, and learning rate of 1e-4. The N-MTLR model produced higher predictive performance including AUC, accuracy, specificity, PPV and NPV than the Cox-regression model for all survival endpoints at the 2-, the 4- and 7-year time points. Both temporal and spatial external validation results indicated that the model had moderate predictive performance and certain clinical application value.

**Conclusions:**

The N-MTLR neural network enables superior nonlinear modeling of BOT recurrence risk, exhibits excellent temporal and spatial generalizability, which supports precise risk stratification for clinical decision-making.

## Introduction

Borderline ovarian tumors (BOTs) represent a distinct category of ovarian neoplasms with unique biological behavior. Histologically, BOTs exhibit epithelial hyperplasia and cellular atypia without stromal invasion, occupying an intermediate position between benign and malignant ovarian tumors (World Health Organization classification: tumors of low malignant potential). Recent epidemiological data indicate an increasing incidence of BOTs, which now account for 14%−15% of primary ovarian neoplasms [1–3]. BOTs primarily affect premenopausal women (30–50 years), with 70%−80% diagnosed at International Federation of Gynecology and Obstetrics (FIGO) stage I, advanced-stage disease remains uncommon [4]. The 5-year survival rate ranges from 95.3% (stage III) to 99.7% (stage I) [5] . Despite this excellent prognosis, recurrence occurs in 5%−20% of cases [6], and 20%−30% of these recurrences exhibit malignant transformation.

This study integrates multiple clinical parameters to develop an accurate predictive model for postoperative BOT recurrence, with particular emphasis on identifying high-risk populations. This approach facilitates risk-stratified follow-up and personalized treatment strategies, potentially improving prognosis while reducing recurrence risk. Consequently, investigating predictors of BOT recurrence and developing robust prediction models have emerged as critical research priorities. Based on comprehensive findings from multiple studies, several factors have been associated with BOT recurrence, including tumor stage, pathological microinvasion, peritoneal implants, micropapillary pattern, postoperative adjuvant chemotherapy, capsule rupture, fertility-sparing surgery (FSS), and laparoscopic surgery [7–9].

Despite the notable recurrence rate, recurrent BOT cases constitute a minority of the overall population, leading to significant class imbalance between recurrent and non-recurrent groups. Class imbalance arises when sample sizes differ significantly between the two categories, with one class being substantially underrepresented. Predictive models trained on imbalanced data typically exhibit classification bias, performing well for the majority class (non-recurrence) but poorly for the minority class (recurrence). The Synthetic Minority Oversampling Technique (SMOTE), introduced by Chawla et al. (2002) [10], represents an innovative approach to addressing class imbalance. This oversampling method generates synthetic samples for the minority class within the feature space, effectively increasing the representation of recurrent cases. By generating synthetic samples through k-nearest neighbor interpolation of minority cases, SMOTE prevents overfitting while enhancing model generalizability [11].

In recent years, the rapid development of artificial intelligence (AI) technologies-particularly deep learning-has provided new technical tools for traditional medical fields to support clinical decision-making. Interdisciplinary research at the intersection of clinical medicine and computer science has emerged as a prominent area, with successful applications in domains such as image classification and computer-aided diagnosis of medical images. Traditional statistical methods describe patterns via mathematical equations, whereas deep learning-owing to its ability to extract hidden features of variables through neural networks and its ease of training-has been widely applied in medical scenarios. For example, deep learning has been used to establish predictive models for lymph node metastasis in bladder cancer patients [12] and to classify breast nodules via artificial neural network analysis of breast ultrasound images [13], providing more efficient, accurate, intelligent solutions for clinicians, patients, and medical institutions. Compared with traditional statistical methods, deep learning can establish non-linear associations between hidden features of variables, thereby further improving the accuracy of data prediction.

This study employs an artificial neural network (ANN)-specifically the Neural Multi-Task Logistic Regression (N-MTLR) model-to predict the probability of postoperative recurrence in BOT patients. The N-MTLR model is a time-varying proportional hazards model centered on deep learning. To capture the time-varying effects of input variables, it constructs a survival function using an ANN with a multilayer perceptron (MLP) core, estimating the probability of an event occurring within different time intervals. The N-MTLR model integrates multiple logistic regression expressions across different time intervals, predicting event probabilities for each interval and replacing the traditional hazard function (conditional failure/mortality rate). This approach avoids the unrealistic proportional hazards assumption in traditional models, thereby enhancing the robustness of predictive analysis.

Given the demonstrated practical utility of deep learning in medical applications, this study proposes an enhanced predictive model for postoperative BOT recurrence by combining the SMOTE algorithm with an ANN. This model aims to identify patients at high risk of postoperative recurrence at an early stage, providing clinicians with a valuable reference for decision-making.

## Materials and methods

### Data sources and participants

Clinical data were collected from patients who met the inclusion criteria at the Department of Obstetrics and Gynecology, Shengjing Hospital of China Medical University, between January 2014 and August 2023. Ethical approval was obtained from the institutional ethics committee of Shengjing Hospital (approval number: 2023PS1207K). For temporal external validation, a cohort of 78 patients from the same hospital (Shengjing Hospital) was included, with data collected between May 2011 and December 2013. For spatial external validation, a cohort of 98 patients from the Second Hospital of Jilin University was used, with data collected between April 2011 and April 2019; ethical approval was obtained from the institutional ethics committee of this hospital (approval number: SY202502062). All participating centers adhered to the core principles for the treatment of BOTs outlined in the National Comprehensive Cancer Network (NCCN) Clinical Practice Guidelines in Oncology: Ovarian Cancer (Version 2025.V1) and the Chinese Expert Consensus on the Diagnosis and Treatment of Borderline Ovarian Tumors (2022 Edition), ensuring the standardization of basic diagnosis and treatment pathways.

Study participants met the following inclusion criteria:

1. Postoperative pathological confirmation of BOT diagnosis;
2. Complete clinicopathological data and follow-up data;
3. Provision of informed consent for telephone follow-up.

Exclusion criteria were:

1. Incomplete clinical data;
2. Refusal to participate in follow-up

### Methods for handling sample imbalance

The SMOTE operates on the principle that adjacent points in the feature space exhibit similar characteristics. Its implementation involves the following steps:

1. Randomly select one sample from the existing recurrent cases, followed by identifying its six nearest neighbors via Euclidean distance calculation;
2. Randomly select one neighbor from these six samples, then calculate the attribute differences between the selected sample and this neighbor;
3. Multiply each attribute difference by a randomly generated value within the range [0, 1], with independent random coefficients assigned to each attribute to ensure sample diversity;
4. Generate synthetic samples by adding the randomized attribute differences to the attributes of the originally selected sample;
5. Repeat steps (1)–(4) iteratively until the desired sample size is achieved.

**Fig. 1:**
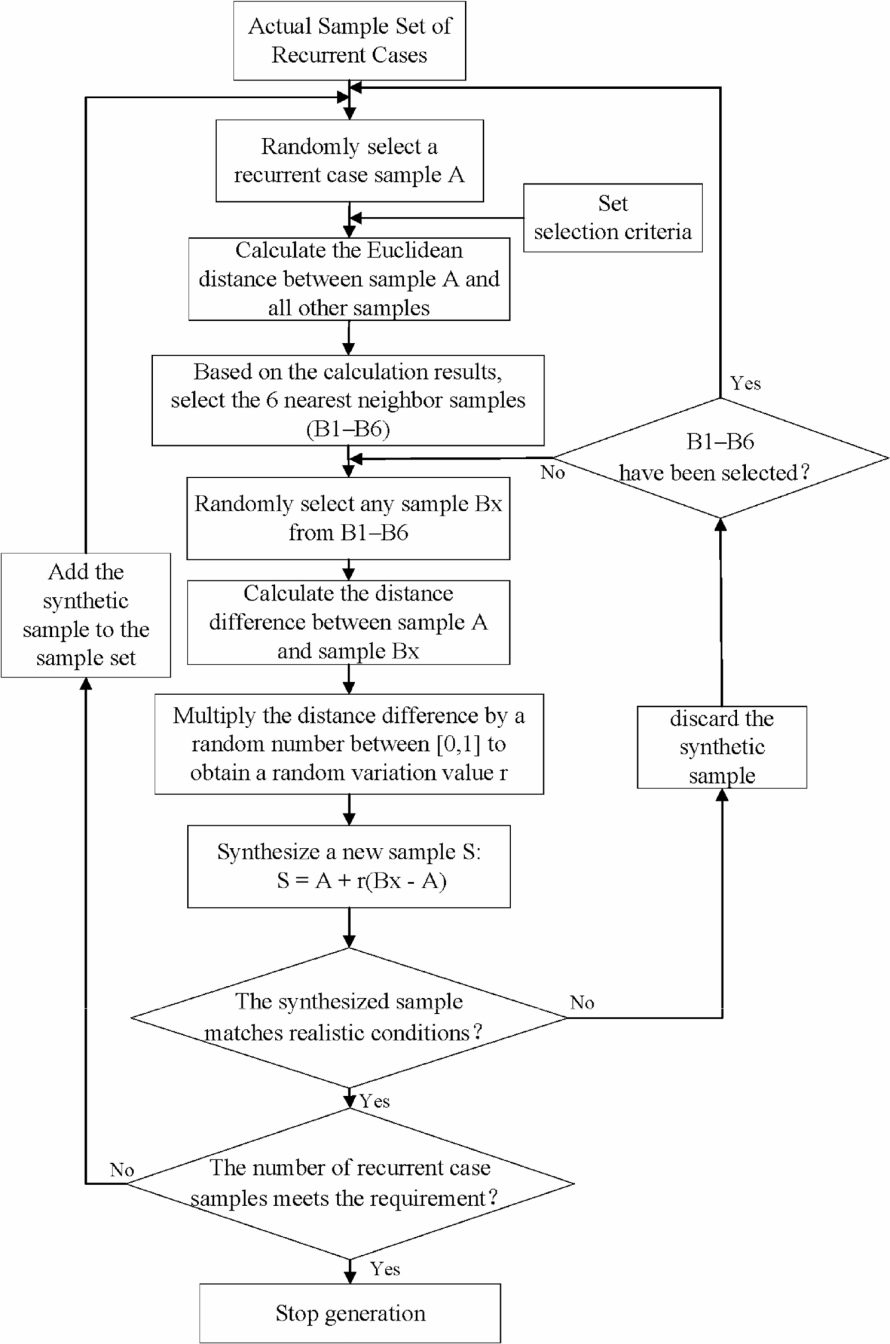
SMOTE synthetic sample flowchart

**Fig. 2:**
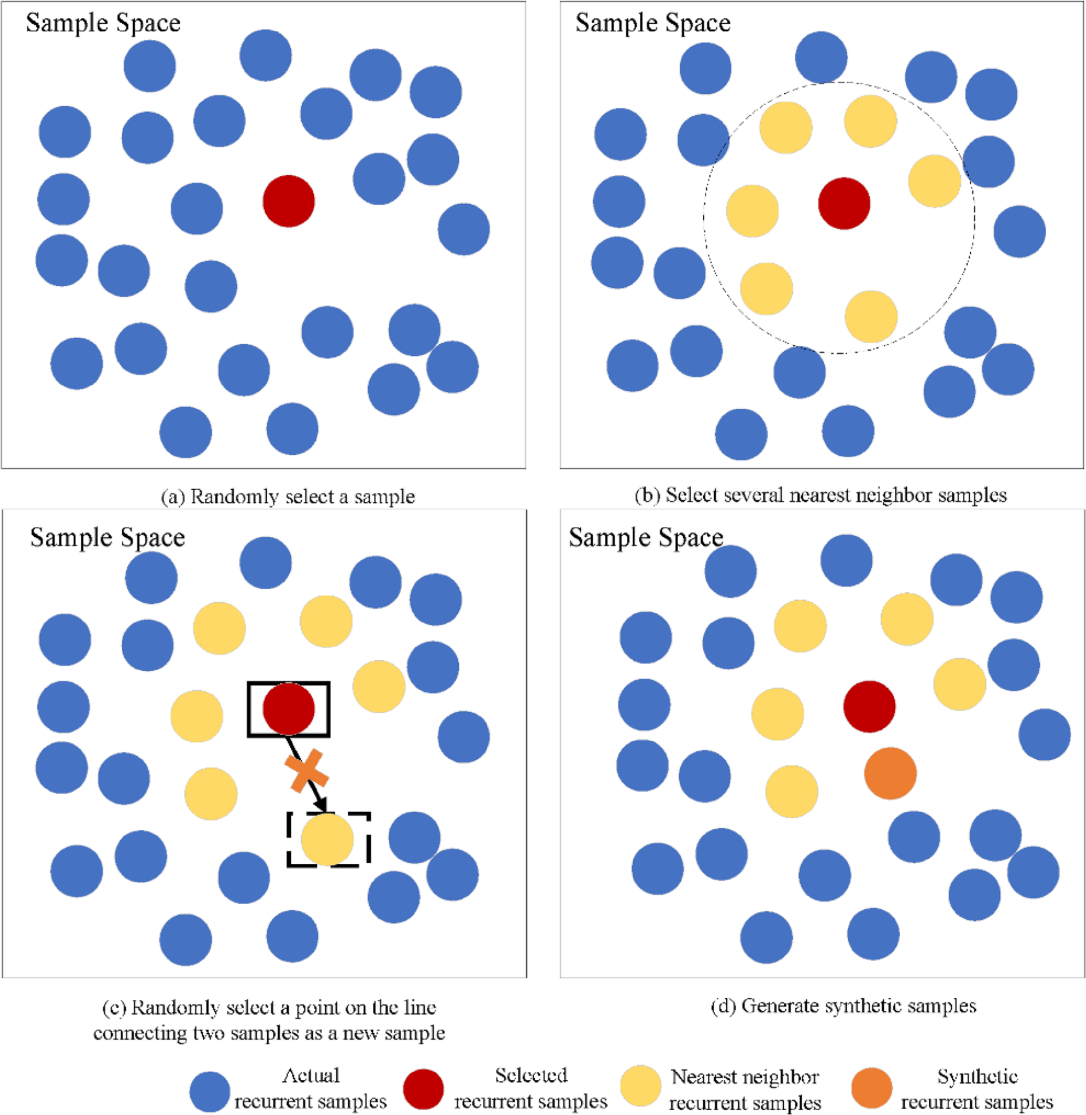
SMOTE Synthetic schematic diagram

Notably, the SMOTE algorithm incorporates previously synthesized samples in subsequent iterations, using them as additional base samples for further synthesis. In our study, the original 76 recurrent cases were augmented with 508 synthetic samples to match the number of non-recurrent cases, resulting in a balanced dataset containing 584 recurrent cases (76 original + 508 synthetic). The flowchart and schematic diagram of this synthesis process are presented in Figs. 1 and 2, respectively.

### Data collection

Data were retrieved from the hospital’s electronic information system and obtained through telephone interviews, covering four major categories:​

1. Patient demographic characteristics, including age, family cancer history, sexual history, parity, menopausal status, comorbidities, presenting symptoms, and smoking and drinking histories.​
2. Preoperative examination results, comprising tumor size (the maximum diameter of the tumor as reported by computed tomography (CT), magnetic resonance imaging (MRI), or ultrasound) and preoperative serum levels of carbohydrate antigen 125 (CA125), carcinoembryonic antigen (CEA), carbohydrate antigen 199 (CA199), alpha - fetoprotein (AFP), carbohydrate antigen 724 (CA724), and human epididymis protein 4 (HE4).​
3. Surgical procedures and postoperative pathological findings, such as the type of primary surgery, surgical approach (laparoscopy or laparotomy), tumor envelope rupture, lesion type, FSS, surgical completeness, performance of omentectomy and lymphadenectomy, histological subtype, micropapillary features, microinfiltration, psammoma bodies and calcification, proportion of the papillary area, peritoneal implantation, ascites cytology, FIGO staging, and whether the tumor is unilateral or bilateral.​
4. Follow - up details on treatment, recurrence, and postoperative fertility obtained via telephone interviews, including postoperative adjuvant therapy, estrogen administration, ovulation induction, and the time to recurrence.

### Follow up

The follow-up period commenced from the surgical date and continued until the earlier of patient death or August 31, 2024. Multiple complementary methods were utilized for follow-up data collection:​

1. A comprehensive review of electronic medical records (EMRs) across hospital information systems and medical imaging archives.​
2. Structured telephone interviews were conducted for patients without clinical follow-up documentation.​

The outcome measures collected included:​

- Survival status (alive or deceased), with the cause of death documented when relevant.​
- Tumor recurrence status, along with the precise recurrence time (recorded to the nearest month).​

Recurrence was defined as the detection of a histologically verified tumor with the same biological characteristics as the primary borderline ovarian tumor (BOT) after complete surgical resection [14]. Clinical confirmation necessitated one of the following:​

1. Pathological verification via biopsy or re-operation of a new ovarian or extragonadal mass.​
2. Consistent suspicious findings on two independent imaging examinations (enhanced CT, enhanced MRI, or PET-CT).​
3. Suspicious imaging findings accompanied by continuously elevated serum CA125 levels.​

The recurrence interval was computed from the date of primary surgery to the date of confirmed recurrence diagnosis.

### Feature dimension reduction and data visualization

Principal component analysis (PCA) is a classical data dimensionality reduction method. Its purpose is to make sure that synthetic samples do not change the overall data distribution of the original dataset. Its essence lies in constructing an orthogonal basis to optimize the projection of high - dimensional data. Initially, the data is mapped to a new coordinate system through linear transformation. Subsequently, according to the principle of dimensionality reduction, it is ensured that the largest variance of the data projection is concentrated on the first coordinate (the first principal component), the second-largest variance is concentrated on the second coordinate (the second principal component), and so on. In this way, the desired dimensionality-reduced features can be selected.​ Furthermore, the t-Distributed Stochastic Neighbor Embedding (t-SNE) method is utilized to visualize the dimensionality-reduced data. This enables the observation and understanding of the dataset’s distribution in the feature space, thus uncovering the relationships among different data points. The data visualization is accomplished using the TSNE function in the sklearn.manifold library of Python.​ t-SNE transforms the similarities between data points into joint probabilities and aims to minimize the Kullback-Leibler divergence (KL divergence) between the joint probabilities of the low-dimensional embedded data and the high-dimensional data. This process mainly consists of two stages:​

First, the high-dimensional data initializes the two-dimensional space variables through the PCA method. Then, the similarities between different data points are iteratively calculated to obtain the probability distribution of the corresponding two-dimensional space variables, thereby maintaining the similarity relationships among the data.​.

Second, it minimizes the KL divergence of the positions of the two-dimensional space variables between the two distributions, guaranteeing that each data point more clearly exhibits its relationship in the original high-dimensional space within the new two- dimensional space.

### Construction of the training set and the internal validation set

The dataset was randomly divided into a training set (70%) and an internal validation set (30%). To validate the equivalence of the distributions between these two sets, appropriate statistical methods were employed to conduct homogeneity tests on all features and outcome labels. A non - significant test result (*P* > 0.05) indicated that both sets were derived from the same underlying population distribution, thus validating the randomization process.

### Establishment of neural multi-task logistic regression model

**Fig. 3:**
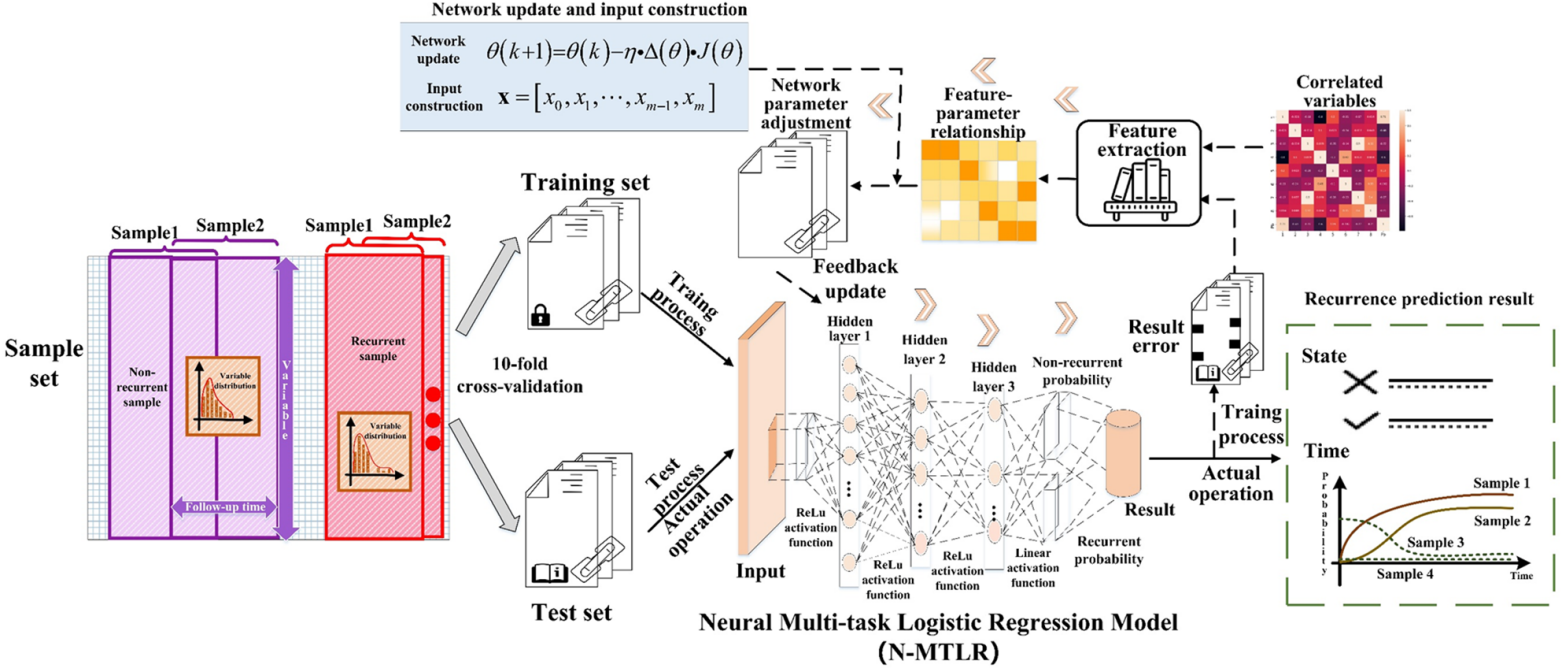
Flowchart of the construction process of multi-task logistic regression model based on neural network

The N-MTLR model is founded upon a neural network architecture consisting of an input layer, three hidden layers, and an output layer. The model-building steps are as follows, as illustrated in Fig. 3:​

**Fig. 4:**
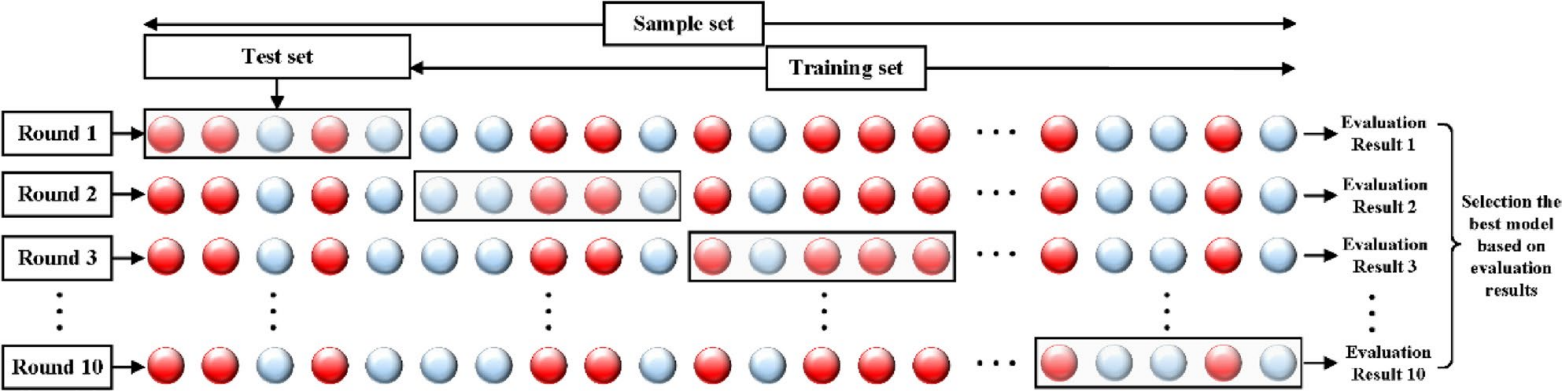
Illustration of the ten-fold cross-validation process

1. Data Preprocessing: Standardize the representation of 34 variables. Specifically, transform the original continuous variables into binary or multi-categorical variables while maintaining the original descriptions of discrete variables unchanged.​
2. Variable Correlation Screening: Employ the Spearman correlation coefficient to screen the correlations among the 34 variable features post-data preprocessing. Spearman correlation coefficient method is chosen to reduce variable redundancy, through the combination of absolute Spearman magnitude and clinical judgment. Through the analysis of feature correlations, variables with high predictive capabilities and interdependencies can be more accurately selected, thereby enhancing the effectiveness and outcomes of feature selection. Based on the criterion of retaining variables with a correlation coefficient greater than 0.5, the variables utilized for constructing the neural network are eventually determined.​
3. Neural Network Construction: To generate recurrence prediction results from input variables, a five layer neural network with a structure of 1 input layer − 3 hidden layers − 1 output layer was constructed. The highly correlated variables obtained in step (2) serve as the input layer and are subsequently fed into the hidden layers. Through the layer by layer feature extraction of neurons in the three hidden layers, the judgment result of the recurrence prediction event and its occurrence probability are ultimately obtained via the output layer.​
4. Neural Network Training: In the Python 3.7 environment, based on the constructed five layer neural network, the glorot_uniform initialization method is adopted to initialize the neural network parameters for weight setting, and the Adam optimization algorithm is utilized to update the neural network parameters. The grid search method is integrated to fine tune the parameters. The neural network parameter update formula is as follows: $\:\theta\:\left(k+1\right)=\theta\:\left(k\right)-\eta\:\cdot\Delta\:\left(\theta\right)\cdot J\left(\theta\right)$. The formula involves performing forward propagation to compute the network outputs and the loss function *J(θ)*, then using backward propagation to calculate the gradient of the loss function with respect to each parameter$\:\:{\Delta\:}\left(\theta\:\right)$. Finally, the parameters *θ* are updated in the direction opposite to the gradient, scaled by the learning rate *η*. This process is repeated iteratively until the model converges. Through multiple iterations, the error is minimized to finally obtain the optimal model parameters. The cross-entropy loss function is chosen to evaluate the model’s performance during the training process and guide the update of model parameters. By minimizing this loss function, the accuracy and generalization ability of the model can be improved.​
5. Network Hyperparameter Adjustment: To prevent deviations in the calculation results due to accidental errors, the study adopted the “cross-validation method” for experimentation. The performance of the N-MTLR model was evaluated through ten-fold cross-validation, and the process of dividing the dataset in the ten-fold cross-validation experiment was shown in Fig. 4. Ten-fold cross-validation involved randomly partitioning the augmented dataset into 10 subsets with approximately equal sample sizes. Sequentially, 9 of these subsets were combined to form the training set, and the remaining 1 subset was used as the validation set. In each trial, diagnostic indicators such as sensitivity and evaluation indicators such as the time-dependent AUC values and Brier scores were calculated. The model’s hyperparameters, including the number of neurons, number of hidden layers, penalty terms (L1 or L2), activation functions, dropout rate, and number of training steps, were manually determined based on empirical experience and kept consistent across all folds to ensure fair and comparable performance evaluations. This consistency ensured that performance metrics were directly comparable, isolating the effect of data variability and enabling robust assessment of the model’s stability for BOT recurrence prediction. The hyperparameter configuration was established prior to cross-validation, and ten-fold cross-validation was used to validate the performance of this fixed configuration.

### Establishment of Cox proportional hazards regression model

The predictive factors initially screened by Lasso regression were subjected to multivariate Cox regression analysis to identify independent risk factors. Subsequently, multivariate Cox regression was used to establish models for predicting the recurrence risk of BOT, respectively. During the model establishment process, the forward stepwise regression method was adopted for variable selection, with the Akaike Information Criterion (AIC) serving as the stopping rule. Meanwhile, by integrating the statistical significance and clinical significance of each indicator, the characteristic variables were finally determined to construct the Cox regression model. Additionally, the “rms” package in R software was applied to establish the prediction model and generate a nomogram.

### Comparison of predictive performances

For the N-MTLR model and the Cox regression model, their predictive performances were evaluated in the validation set using the time-dependent area under the curve (tdAUC), time-dependent Brier Scores (td-Brier Scores), accuracy, sensitivity, specificity, positive predictive value (PPV), and negative predictive value (NPV). A larger tdAUC value indicates a stronger ability of the model to predict the recurrence probability of BOT. Generally, tdAUC values are interpreted as follows: 0.5–0.7: indicates the model has a moderate predictive effect; 0.7–0.9: indicates the model has a good predictive effect; 0.9: indicates the model has an excellent predictive effect. The td-Brier Scores range from 0 to 1, where values closer to 0 indicate superior calibration of the model.

### Performance evaluation of the neural multi-task logistic regression model

The predictive performance of the N-MTLR model was evaluated on the internal validation set, temporal external validation set, and spatial external validation set, respectively, using diagnostic efficacy indicators including accuracy, sensitivity, specificity, PPV, NPV, td-AUC, and td-Brier Scores.

### Statistical analysis

Continuous variables were expressed as mean ± standard deviation (for normally distributed data) or median (interquartile range) (for skewed data), while categorical variables were presented as counts. For tumor markers-including CA125, CEA, CA199, AFP, CA724, and HE4-their continuous values were converted into categorical variables based on their respective clinical normal reference ranges. For continuous variables, the Kolmogorov-Smirnov test was first conducted to assess the normality of distribution. When comparing means between two independent samples, the independent samples t-test was used if the data followed a normal distribution; in contrast, the Mann-Whitney U test was applied if the data exhibited a skewed distribution. For categorical variables, comparisons were performed using the chi-square test or Fisher’s exact test (the latter was used when the expected frequency of any cell was < 5, to ensure statistical validity). All tests were two-tailed, and a *P*-value < 0.05 was considered statistically significant. Statistical analyses were performed using SPSS 26.0 (IBM Corporation, Armonk, NY, USA), R software 4.3.1 (R Foundation for Statistical Computing, Vienna, Austria), and Python 3.7.16 (Python Software Foundation, Wilmington, DE, USA).

## Results

### Patient characteristics

A total of 660 patients with BOTs were included in this retrospective study. Among these patients, 76 with recurrence were assigned to the recurrence group, and the remaining 584 without recurrence were assigned to the non-recurrence group, with a group ratio of approximately 1:7.7. To address this significant class imbalance, the SMOTE algorithm was applied to generate synthetic recurrence cases, until balanced class representation was achieved. The final analytic cohort comprised the following components: Original cases: 660 patients (584 non-recurrence and 76 recurrence); Synthetic cases: 508 patients (generated from the original recurrence cases); Final balanced dataset: 1168 patients (584 patients per group).

**Fig. 5:**
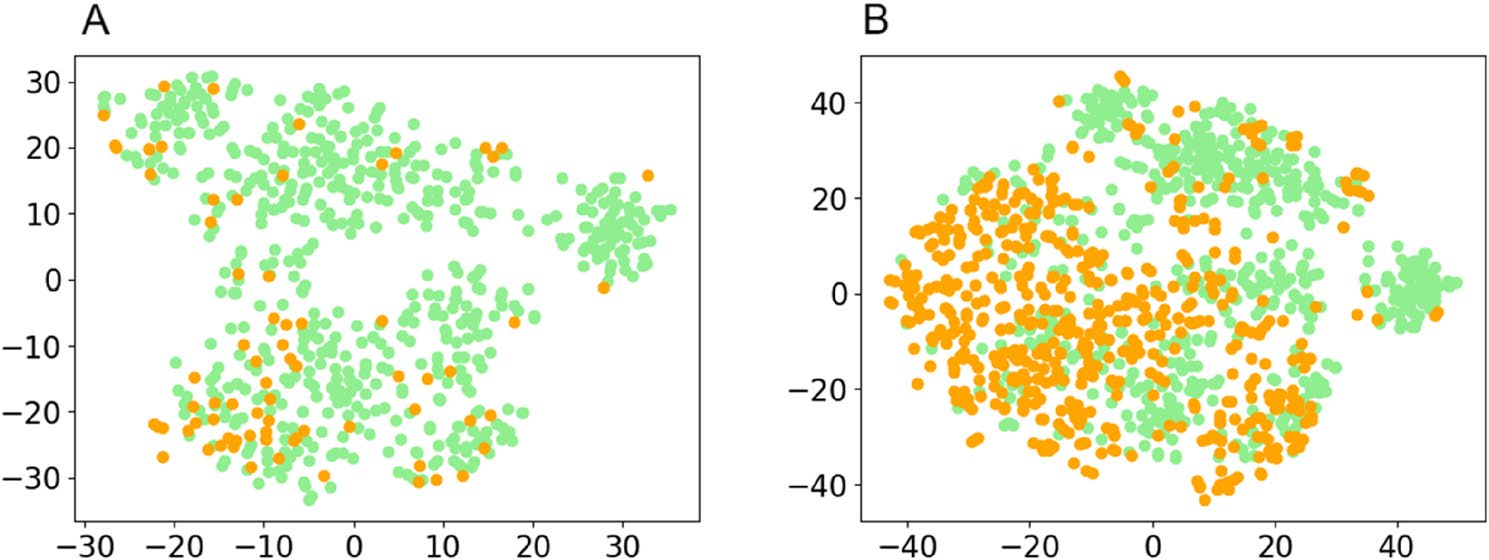
Principal component analysis of the distribution of data in the original dataset and the balanced dataset

PCA was employed for initial dimensionality reduction of the data. Subsequently, the similarity between the dimensionality-reduced data points was further converted into the joint probability among the data, and the KL divergence among the data was minimized. This minimization ensured that similar data points were localized to adjacent regions in the low-dimensional data space. The distribution of the two patient groups’ data was visualized using the t-distributed Stochastic Neighbor Embedding (t-SNE) method (Fig. 5), where green points represented non-recurrence patients and yellow points represented recurrence patients, respectively. Fig. 5(A) presents the data distribution of the original dataset. As observed in this figure, the proportion of non-recurrence patients is substantially higher than that of recurrence patients-a distribution that may lead the recurrence prediction model to reference the analytical results of non-recurrence patients. Fig. 5(B) illustrates the data distribution of the balanced dataset: based on the original dataset, the SMOTE method was used to ensure that different types of data were distributed in adjacent regions of the low-dimensional data space. From the visualized data distributions in Fig. 5, the two groups exhibit similar data distributions, and the distribution of the original dataset has not been significantly altered. This observation confirms the feasibility and rationality of the data augmentation approach.

Table 1 presents the basic clinical characteristics of subjects in the recurrence and non-recurrence groups. The results showed that the clinical characteristics with statistical significance between the two groups were consistent between the balanced dataset and the original dataset. The dataset was randomly split into a training set (70%, 817 cases) and an internal validation set (30%, 351 cases). In the training set, the number of non-recurrence cases and recurrence cases was 404 and 413, respectively, yielding a ratio of 0.98:1; In the internal validation set, the number of non-recurrence cases and recurrence cases was 180 and 171, respectively, with a ratio of 1.05:1. To ensure the training set and internal validation set had identical sample distributions, a homogeneity test of distributions was performed on all features and labels of the two sets. The results indicated no statistical differences across the various clinical characteristics, confirming that the samples in the training set and internal validation set were derived from the same population.

**Table 1:** Clinical characteristics of the non-recurrence group, recurrence group, training set and validation set in the balanced dataset

Characteristics	Classifications	Total (*n* = 1168)		Non-recurrence group(*n* = 584)	Recurrence group (*n* = 584)	*P*	Training set (*n* = 817)	Validation set (*n* = 351)	*P*
Mean age at diagnosis (years old)		36.66 ± 12.82		43.71 ± 14.68	32.99 ± 9.97	0.000^a^	37.73 ± 13.16	38.97 ± 13.51	0.143^a^
Family cancer history	Yes	44		26	18	0.219	25	19	0.053
	No	1124		558	566		792	332	
Sexual history	Yes	1068		538	530	0.403	749	319	0.657
	No	100		46	54		68	32	
Parity	Yes	654		411	243	0.000	449	205	0.277
	No	514		173	341		368	146	
Menopausal status	Yes	239		194	45	0.000	156	83	0.077
	No	929		390	539		661	268	
Comorbidities	Yes	195		107	88	0.052	117	62	0.146
	No	1109		525	584		700	289	
Presenting symptom	Abdominal pain	419		292	127	0.000	286	133	0.626
	Medical examination	683		243	440		485	198	
	Abnormal bleeding	66		49	17		46	20	
Smoking and drinking history	Yes	48		27	21	0.376	36	12	0.436
	No	1120		557	563		781	339	
Surgical route	Laparotomy	800		439	361	0.000	549	251	0.146
	Laparoscope	368		145	223		268	100	
Tumor envelope rupture	No	914		482	432	0.000	644	270	0.470
	Yes	254		102	152		173	81	
Fertility-sparing surgery	No	419		349	70	0.000	293	126	0.991
	Yes	749		235	514		524	225	
Completeness of surgery	No	680		256	424	0.000	483	197	0.342
	Yes		488	328	160		334	154	
Omentectomy	No		650	253	397	0.000	459	191	0.578
	Yes		518	331	187		358	160	
Lymphadenectomy	No		782	317	465	0.000	558	224	0.136
	Yes		386	267	119		259	127	
Histological subtype	Serous		715	317	398	0.000	513	202	0.055
	Mucinous		247	141	106		175	72	
	Seromucous		111	53	58		73	38	
	Others		95	73	22		56	39	
Micropapillary	No		1081	547	534	0.147	755	326	0.781
	Yes		87	37	50		62	25	
Microinfiltration	No		1130	580	550	0.000	790	340	0.880
	Yes		38	4	34		27	11	
Psammoma bodies and calcification	No		1139	573	566	0.188	794	345	0.265
	Yes		29	11	18		23	6	
Type of lesion	Exogenous tumor		56	9	47	0.000	44	12	0.149
	Endogenous tumor		1112	575	537		773	339	
Peritoneal implantation	No		1054	550	504	0.000	732	322	0.258
	Yes		114	34	80		85	29	
Proportion of papillary area (%)			46.81 ± 28.10	46.18 ± 28.67	49.35 ± 25.57	0.103^a^	46.19 ± 28.70	49.31 ± 25.45	0.108^a^
Ascites cytology	No		1059	507	552	0.000	736	323	0.297
	Yes		109	77	32		81	28	
FIGO staging	I		944	514	430	0.000	661	283	0.914
	II		133	24	109		94	39	
	III		91	46	45		62	29	
Is the tumor unilateral or bilateral	Unilateral		819	443	376	0.000	576	243	0.663
	Bilateral		349	141	208		241	108	
Tumor size (cm)			9.99 ± 5.73	11.25 ± 6.46	8.28 ± 4.33	0.000^a^	9.97 ± 5.71	10.06 ± 5.80	0.808^a^
CA125	0–35 U/mL		473	236	237	0.952	331	142	0.985
	> 35 U/mL		695	348	347		486	209	
CEA	0–5 ng/mL		1100	543	557	0.080	770	330	0.878
	> 5 ng/mL		68	41	27		47	21	
CA199	0–37 U/mL		947	446	501	0.000	668	279	0.363
	> 37 U/mL		221	138	83		149	72	
AFP	0–9 ng/mL		1153	574	579	0.194	806	347	0.774
	> 9 ng/mL		15	10	5		11	4	
CA724	0–6.9 U/mL		900	457	443	0.330	629	271	0.935
	> 6.9 U/mL		268	127	141		188	80	
HE4	0–140 pmol/L		1070	526	544	0.057	749	321	0.899
	> 140 pmol/L		98	58	40		68	30	
Estrogen	No		1133	554	579	0.000	790	343	0.346
	Yes		35	30	5		27	8	
Ovulation induction	No		1112	576	536	0.000	779	333	0.726
	Yes		56	8	48		38	18	
Postoperative adjuvant therapy	No		986	482	504	0.076	688	298	0.766
	Yes		182	102	80		129	53	

*FIGO* International Federation of Gynecology and Obstetrics

*P*-value not indicated: chi-square test

^a^Independent sample t-test

### Cox model variable selection and development

**Fig. 6:**
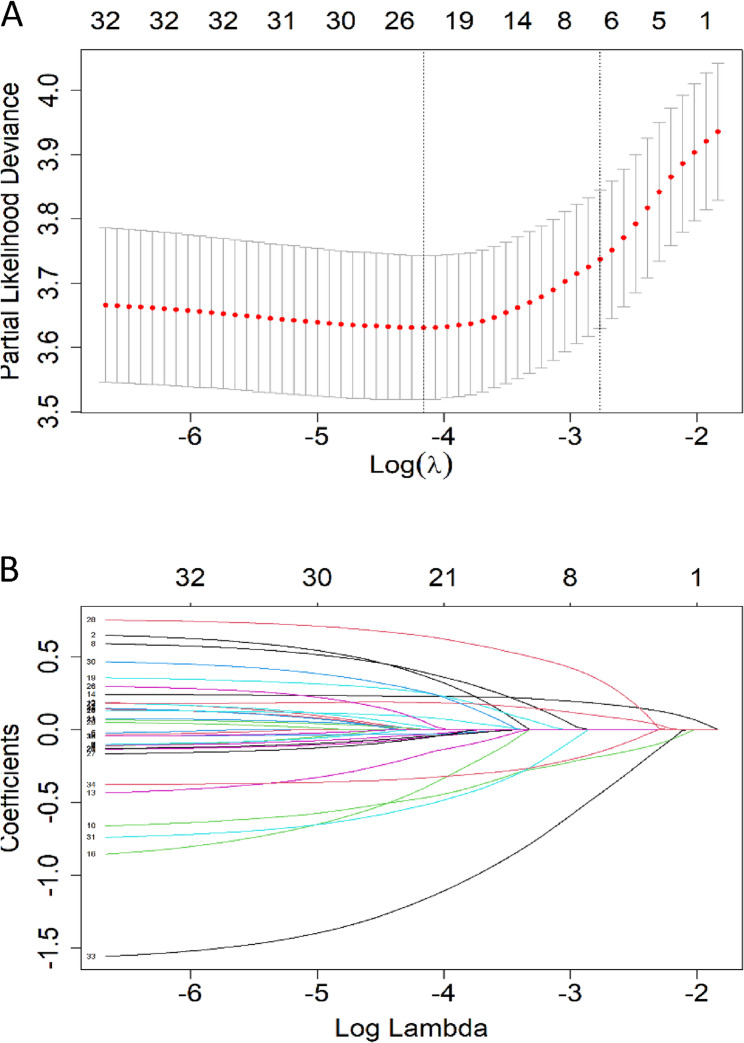
Identification of recurrence-related feature variables of BOT via Lasso regression (**A)** Cross-validation plot of Lasso regression. The penalty value was determined based on the lowest point of the curve; (**B**) Coefficient distribution plot of Lasso regression. The x-axis represents log lambda, and the y-axis denotes Lasso regression coefficients

**Fig. 7:**
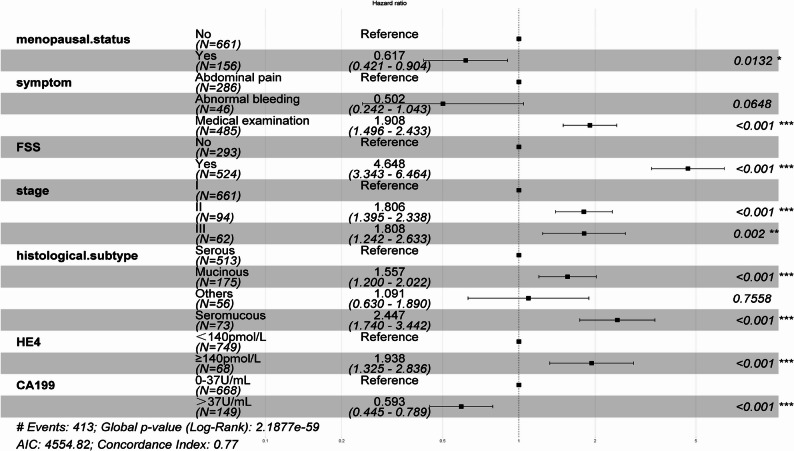
Forest plot of risk factors for postoperative recurrence in BOT patients in the training set

**Fig. 8:**
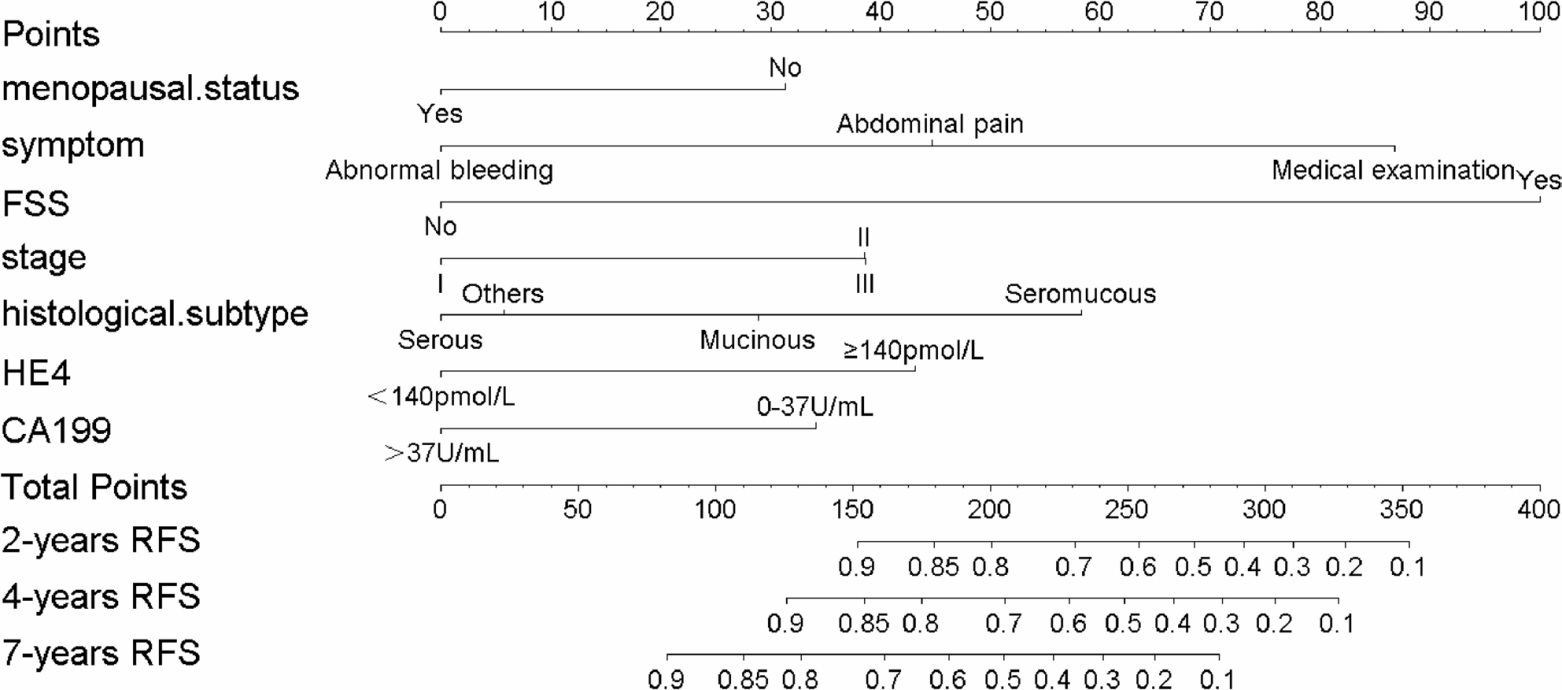
Nomogram of the postoperative recurrence prediction model for BOT patients in the training set

In the training set, lasso regression was performed for variable selection (Fig. 6). Results of this regression indicated that the following factors were significantly associated with the recurrence of BOTs: sexual activity history, menopausal history, clinical symptoms, surgical approach, pathological type, clinical stage, performance of comprehensive staging surgery, omentectomy status, presence of peritoneal implants, ascites cytology results, unilateral/bilateral tumor, CA199, HE4, CEA, tumor diameter, ovulation induction history, lesion type, and papillary area. Subsequently, a Cox regression model was constructed via multivariate Cox analysis (Table 2) to identify independent risk factors for BOT recurrence. These independent risk factors included menopausal history, clinical symptoms, performance of fertility-sparing surgery, pathological type, clinical stage, CA199 level, and HE4 level. The results of the multivariate analysis were visualized using a forest plot (Fig. 7). Finally, a nomogram was developed to establish a predictive model for estimating the 2-year, 4-year, and 7-year post-surgical recurrence-free survival rates (Fig. 8).

**Table 2:** Multivariate Cox analyses of risk factors for BOT recurrence in the training set

Variable	Category	Multivariate analysis	
		HR (95%CI)	*P*
Menopausal status	No	1	0.019
	Yes	0.63(0.43–0.9)	
Presenting symptom	Abdominal pain	1	
	Medical examination	1.90(1.49–2.42)	< 0.001
	Abnormal bleeding	0.50(0.24–1.04)	0.064
Fertility-sparing surgery	No	1	< 0.001
	Yes	4.65(3.34–6.47)	
Histological subtype	Serous	1	
	Mucinous	1.56(1.20–2.02)	0.001
	Seromucous	2.45(1.74–3.44)	< 0.001
	Others	1.18(0.68–2.04)	0.561
FIGO staging	I	1	
	II	1.81(1.40–2.34)	< 0.001
	III	1.81(1.24–2.63)	0.002
CA199	0–37 U/mL	1	
	> 37 U/mL	0.59(0.44–0.78)	< 0.001
HE4	<140 pmol/L	1	
	≥ 140 pmol/L	1.94(1.32–2.83)	< 0.001

### Correlation-based variable selection

**Fig. 9:**
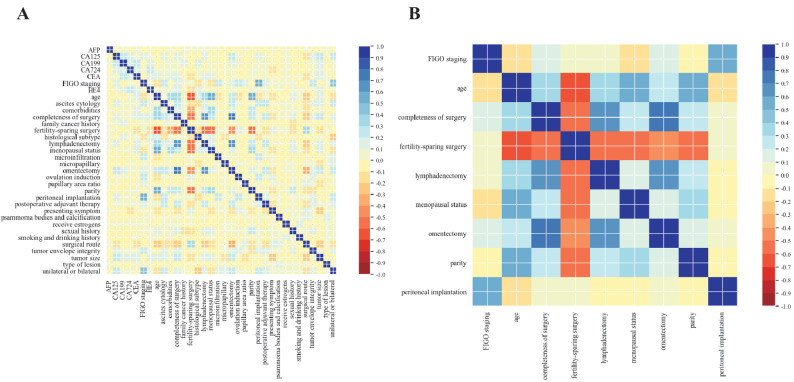
Correlation screening of variables for patients with BOT in the training set, 9 variables with a correlation of more than 0.5 were screened for the construction of the neural multi-task logistic regression model

Correlation screening was conducted for the 34 variables in the training set (Fig. 9). All selected variables are implemented by a rigorous preprocessing procedure to ensure they were in a standardized numerical format suitable for training. The categorical variables were transformed into a machine-readable format using one-hot encoding, this process converts each categorical feature into separate binary columns, allowing the neural network to interpret the data. The continuous variable “age” is divided into two categories-“≤ 40 years old” and “>40 years old”-based on its median. Following the above preprocessing steps, the input matrix was constructed as a two-dimensional matrix. Wherein, row and column of input matrix represent patient encoded features of the selected variables, respectively. In the correlation matrix, the values represent the correlation coefficients between pairs of variables, and the color intensity reflects the strength of the correlation: dark blue indicates a strong positive correlation, while dark red denotes a strong negative correlation. Subsequently, nine variables were selected for the construction of the neural network model, including FSS, comprehensive staging surgery, comorbidities, FIGO staging, age, omentectomy status, lymphadenectomy status, parity, menopausal status, and presence of peritoneal implants.

### Development of the neural multi-task logistic regression model

**Fig. 10:**
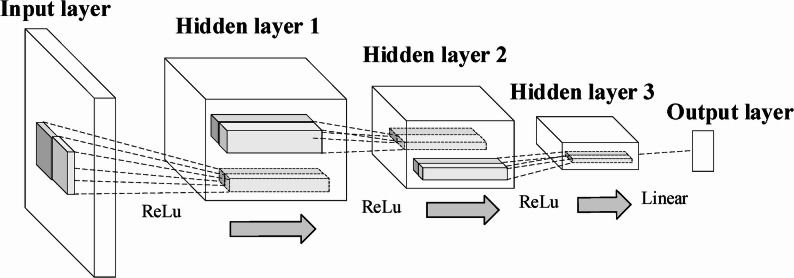
Structure of the neural multi-task logistic regression model for predicting BOT recurrence, a 5-layer neural network with 1 input layer, 3 hidden layer and output layer is structured, with Rectified Linear Unit (ReLU) activation

**Fig. 11:**
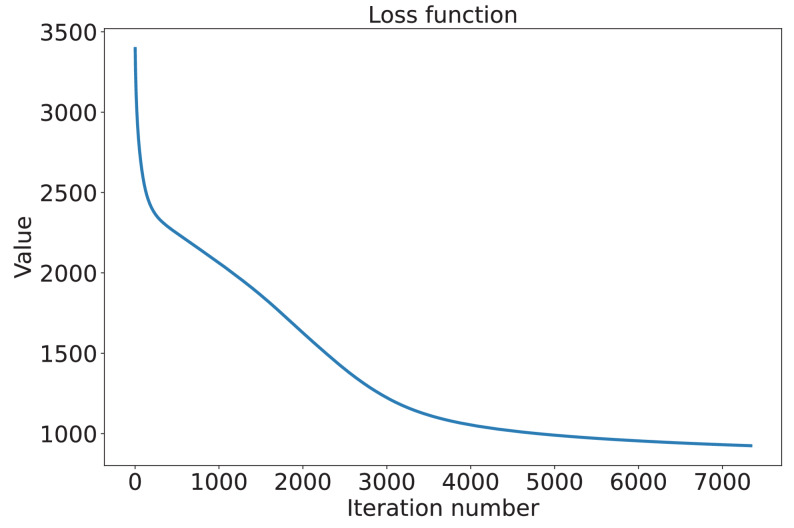
Loss function value. It is used to measure the performance of the model in the training process, guide the updating of model parameters, and improve the accuracy and generalization ability of the model by minimizing the loss function

The N-MTLR model was a neuron-based architecture consisting of five functional layers: one input layer, three hidden layers (with 128, 64, and 32 neurons, respectively; totaling 224 neurons), and one output layer (Fig. 10). The Adam optimizer was used for parameter updates, while L2 regularization and Dropout techniques were applied to mitigate overfitting. To evaluate model performance, ten-fold cross-validation was conducted to maximize the value of the limited clinical dataset and to ensure that the model’s performance during both training and validation was robust (Table 3). The model’s hyperparameters, including the learning rate, number of network layers, number of neurons per layer, activation functions, regularization strength, and optimization algorithm, were manually determined based on empirical experience and kept consistent across all folds to ensure fair and comparable performance evaluations.This consistency ensured that performance metrics were directly comparable, isolating the effect of data variability and enabling robust assessment of the model’s stability for BOT recurrence prediction. Throughout the ten-fold cross-validation process, the hyperparameter configuration was kept identical across all folds, including the learning rate, the number of network layers, the number of neurons per layer, activation functions, regularization strength, and the optimization algorithm. A comprehensive analysis of the evaluation metrics revealed that the first training configuration achieved the optimal performance for BOT recurrence prediction. The optimal parameters of the final model were as follows: glorot_uniform initialization, a 30% Dropout rate, an L2 regularization parameter of 1e-2, and a learning rate of 1e-4. As presented in Fig. 11, the loss function value of the N-MTLR model decreased from 3462 to 928 after 7,340 iterations.

**Table 3:** Ten-fold cross-validation results of the N-MTLR model (2-/4-/7-year)

Test set	Sensitivity	Specificity	PPV	NPV	Accuracy	AUC	Brier score
1	0.78/0.78/0.75	0.80/0.80/0.77	0.79/0.79/0.76	0.79/0.79/0.76	0.79/0.79/0.76	0.80/0.80/0.77	0.10/0.09/0.15
2	0.75/0.81/0.78	0.77/0.82/0.80	0.76/0.81/0.79	0.76/0.81/0.79	0.76/0.81/0.79	0.77/0.84/0.80	0.08/0.09/0.14
3	0.76/0.78/0.77	0.78/0.80/0.79	0.77/0.79/0.78	0.77/0.79/0.78	0.77/0.79/0.78	0.78/0.80/0.79	0.10/0.10/0.16
4	0.76/0.79/0.77	0.78/0.80/0.79	0.77/0.79/0.78	0.77/0.79/0.78	0.77/0.79/0.78	0.78/0.81/0.79	0.08/0.09/0.15
5	0.77/0.78/0.76	0.79/0.80/0.78	0.78/0.79/0.77	0.78/0.79/0.77	0.78/0.79/0.77	0.79/0.80/0.78	0.08/0.08/0.15
6	0.77/0.77/0.76	0.79/0.79/0.78	0.78/0.78/0.77	0.78/0.78/0.77	0.78/0.78/0.77	0.79/0.79/0.78	0.11/0.10/0.15
7	0.78/0.78/0.78	0.80/0.80/0.80	0.79/0.79/0.79	0.79/0.79/0.79	0.79/0.79/0.79	0.80/0.80/0.80	0.09/0.09/0.17
8	0.79/0.79/0.76	0.80/0.80/0.78	0.79/0.79/0.77	0.79/0.79/0.77	0.79/0.79/0.77	0.81/0.81/0.78	0.11/0.10/0.16
9	0.77/0.80/0.77	0.79/0.81/0.79	0.78/0.80/0.78	0.78/0.80/0.78	0.78/0.80/0.78	0.79/0.82/0.79	0.08/0.08/0.15
10	0.81/0.79/0.77	0.82/0.80/0.79	0.81/0.79/0.78	0.81/0.79/0.78	0.81/0.79/0.78	0.83/0.81/0.79	0.07/0.07/0.14

*PPV* Positive predictive value, *NPV* Negative predictive value

### Comparison of predictive performances

The N-MTLR model produced higher predictive performance including AUC, accuracy, specificity, PPV and NPV than the Cox-regression model for all survival endpoints at the 2-, the 4- and 7-year time points (Table 4).

**Table 4:** Prognostic accuracies of the N-MTLR model and the Cox-regression model

Time point	Model	tdAUC	td-Brier scores	Accuracy	Sensitivity	Specificity	PPV	NPV
2-year	COX	0.78	0.10	0.75	0.78	0.71	0.73	0.76
		[0.73,0.83]	[0.07,0.13]	[0.70,0.80]	[0.72,0.84]	[0.64,0.78]	[0.68,0.78]	[0.71,0.82]
	N-MTLR	0.80	0.10	0.83	0.76	0.92	0.88	0.79
		[0.75,0.85]	[0.07,0.13]	[0.79,0.87]	[0.70,0.82]	[0.90,0.94]	[0.84,0.93]	[0.75,0.84]
4-year	COX	0.78	0.09	0.76	0.77	0.75	0.76	0.77
		[0.73,0.83]	[0.06,0.12]	[0.71,0.81]	[0.71,0.83]	[0.68,0.81]	[0.71,0.81]	[0.72,0.82]
	N-MTLR	0.80	0.09	0.84	0.80	0.89	0.88	0.82
		[0.75,0.85]	[0.06,0.12]	[0.81,0.88]	[0.74,0.85]	[0.84,0.93]	[0.83,0.92]	[0.77,0.86]
7-year	COX	0.75	0.12	0.75	0.73	0.74	0.74	0.73
		[0.70,0.80]	[0.09,0.15]	[0.69,0.79]	[0.67,0.79]	[0.67,0.81]	[0.69,0.79]	[0.68,0.78]
	N-MTLR	0.77	0.15	0.76	0.74	0.81	0.79	0.76
		[0.72,0.82]	[0.11,0.19]	[0.73,0.82]	[0.68,0.80]	[0.75,0.87]	[0.74,0.85]	[0.71,0.81]

### Construction of the Temporal external validation dataset

A total of 78 cases were included in the temporal external validation dataset. During follow-up, 10 cases experienced recurrence, while 68 remained recurrence-free. The following variables were extracted for each patient: FSS, comprehensive staging surgery, comorbidities, FIGO staging, age, omentectomy, lymphadenectomy, parity, menopausal status, and peritoneal implantation. Temporal external validation was performed using these variables to assess the model’s temporal generalizability (Table 5).

**Table 5:** Clinical characteristics of N-MTLR model variables in the Temporal external validation set

Characteristics	Classifications	Total Population (m = 78)	Non-recurrence Group(*N* = 68)	Recurrence Group(*N* = 10)
Mean age at diagnosis (years old)		41.40 ± 12.98^a^	41.72 ± 13.39^a^	39.20 ± 10.00^a^
Parity	Yes	52	47	5
	No	26	21	5
Menopausal status	Yes	19	18	1
	No	59	50	9
Fertility-sparing surgery	No	44	43	1
	Yes	34	25	9
Completeness of surgery	No	25	20	5
	Yes	53	48	5
Omentectomy	No	25	20	5
	Yes	53	48	5
Lymphadenectomy	No	27	21	6
	Yes	51	47	4
Peritoneal implantation	No	74	64	10
	Yes	4	4	0
FIGO staging	I	69	59	10
	II	4	4	0
	III	5	5	0

^a^x ± s; unlabeled: n

### Construction of the Spatial external validation dataset

A total of 98 cases were included in the spatial external validation dataset, with 11 recurrent cases and 87 non-recurrent cases. The following nine variables were extracted for model validation: FSS, comprehensive staging surgery, comorbidities, FIGO staging, age, omentectomy, lymphadenectomy, parity, menopausal status, and peritoneal implantation. Spatial external validation was performed using these variables to evaluate the model’s geographic generalizability (Table 6).

**Table 6:** Clinical characteristics of N-MTLR model variables in the Spatial external validation set

Characteristics	Classifications	Total Population (*N* = 78)	Non-recurrence Group (*N* = 68)	Recurrence Group (*N* = 10)
Mean age at diagnosis (years old)		32.20 ± 9.90^a^	32.55 ± 9.95^a^	29.45 ± 9.51^a^
Parity	Yes	85	78	7
	No	13	9	4
Menopausal status	Yes	17	17	0
	No	81	70	11
Fertility-sparing surgery	No	18	17	1
	Yes	80	70	10
Completeness of surgery	No	83	73	10
	Yes	15	14	1
Omentectomy	No	73	64	9
	Yes	14	12	2
Lymphadenectomy	No	92	82	10
	Yes	6	5	1
Peritoneal implantation	No	94	84	10
	Yes	4	3	1
FIGO staging	I	81	75	6
	II	11	8	3
	III	6	4	2

a: x ± s; unlabeled: n

### Model evaluation of N-MTLR based on validation set

The N-MTLR model showed high predictive performances in terms of accuracy, specificity, PPV, and NPV (Table 7) in the internal validation set, temporal external validation set and spatial external validation Set.

**Table 7:** General diagnostic indicators of the N-MTLR model in the validation set

Validation Set	Accuracy	Sensitivity	Specificity	PPV	NPV
Internal validation set (2-/4-/7-year)	0.83/0.84/0.76	0.76/0.80/0.74	0.92/0.89/0.81	0.88/0.88/0.79	0.79/0.82/0.76
Temporal External Validation Set (2-/4-/7-year)	0.72/0.73/0.74	0.53/0.82/0.82	0.91/0.64/0.67	0.86/0.69/0.67	0.66/0.79/0.82
Spatial External Validation Set (2-/4-/7-year)	0.67/0.66/0.67	0.82/0.81/0.90	0.64/0.63/0.65	0.63/0.62/0.63	0.97/0.96/0.98

*PPV* Positive predictive value, *NPV* Negative predictive value

**Fig. 12:**
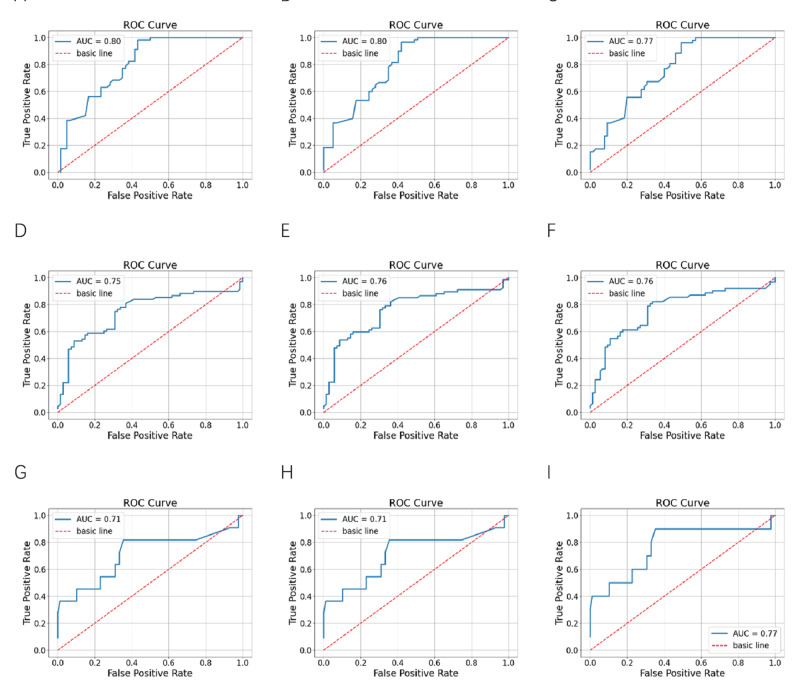
The time-dependent ROC curves of the postoperative recurrence prediction models constructed based on neural networks for the internal validation set, temporal external validation set and spatial external validation set

The AUC values of this model in the internal validation set, the temporal external validation set, and the spatial external validation set at 2 years, 4 years, and 7 years after the operation were all above 0.7 (Fig. 12), it can predict well whether the patient relapsed at each time point, indicating that the NMTLR model has good discrimination.

Internal validation set A-C: 2-year postoperative ROC curve (AUC = 0.80); 4-year postoperative ROC curve (AUC = 0.80); 7-year postoperative ROC curve (AUC = 0.77); Temporal external validation set D-F: 2-year postoperative ROC curve (AUC = 0.75); 4-year postoperative ROC curve (AUC = 0.76); 7-year postoperative ROC curve (AUC = 0.76); Spatial External validation set G-I: 2-year postoperative ROC curve (AUC = 0.71); 4-year postoperative ROC curve (AUC = 0.71); 7-year postoperative ROC curve (AUC = 0.77).

**Fig. 13:**
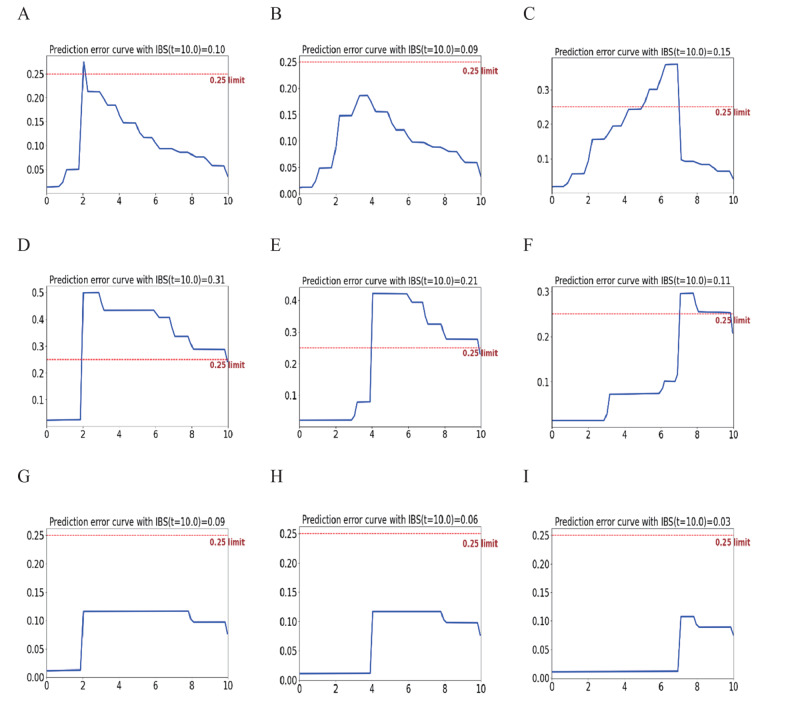
The Brier scores of the postoperative recurrence prediction models constructed based on neural networks for the internal validation set, temporal external validation set and spatial external validation set Internal validation set **A**-**C** 2-year Brier scores (IBS = 0.10); 4-year Brier scores (IBS = 0.09); 7-year Brier scores (IBS = 0.15); Temporal external validation set **D**-**F** 2-year Brier scores (IBS = 0.31); 4-year Brier scores (IBS = 0.21); 7-year Brier scores (IBS = 0.11); Spatial External validation set **G**-**I** 2-year Brier scores (IBS = 0.09); 4-year Brier scores (IBS = 0.06); 7-year Brier scores (IBS = 0.03)

The IBS of the internal validation set, the temporal external validation set and the spatial external validation set at 2 years, 4 years and 7 years after the operation are shown in Fig. 13. Except the Brier score of the temporal external validation set at 2 years after the operation, the score values of this model were all less than 0.25, and the prediction accuracy of this model was relatively high.

### Analysis of the importance of rearranging characteristic variables in the N-MTLR model

**Fig. 14:**
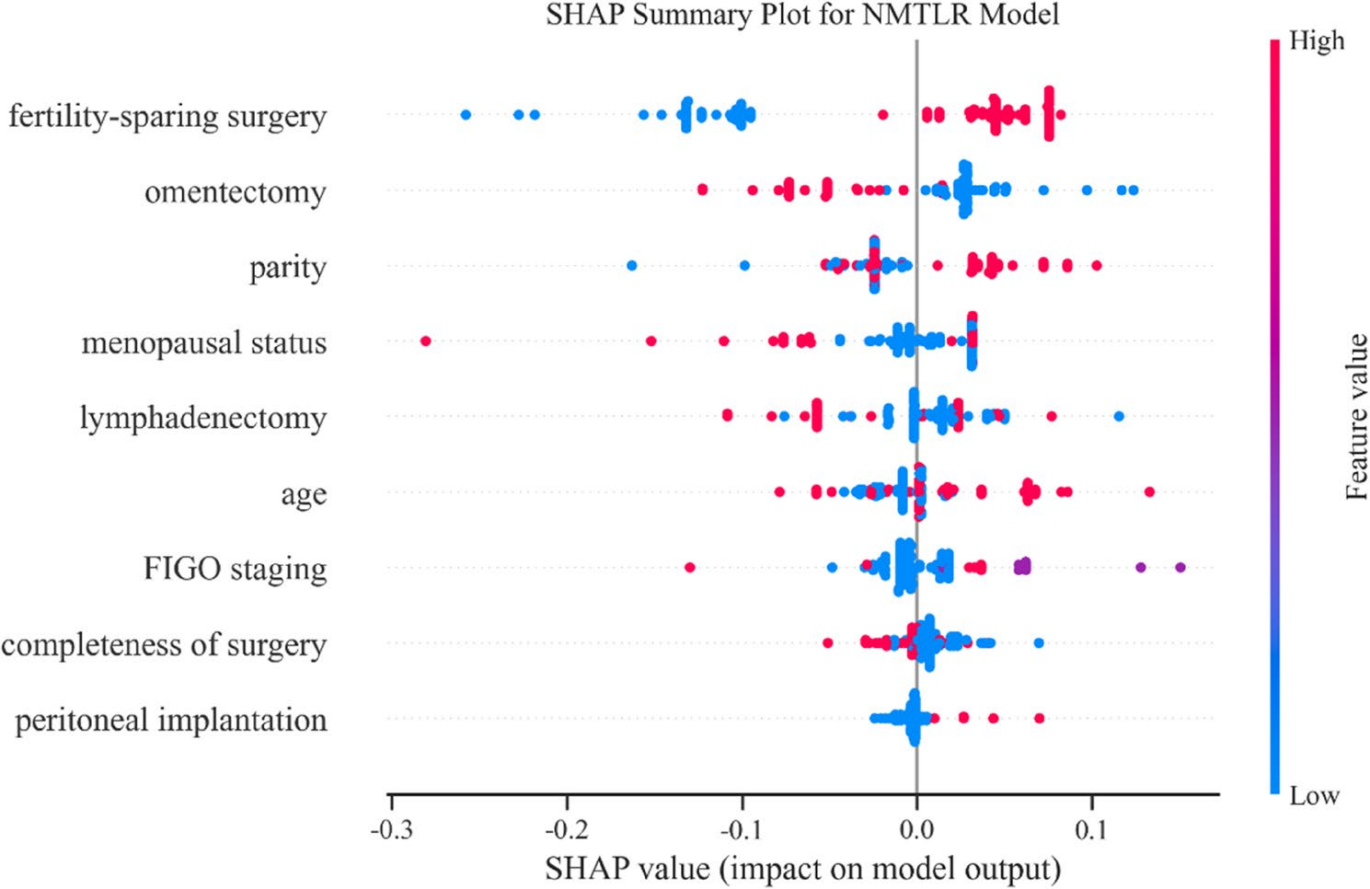
SHAP analysis results in the training set

Since the N-MTLR model is a nonlinear black box model-distinct from traditional regression models-the relationship between its input variables and prognosis cannot be directly inferred. In this study, the SHapley Additive exPlanations (SHAP) was employed to assess the impact of each variable on model performance in the training set, which indirectly reflects the association between variables and prognosis. Fig. 12 demonstrates the corresponding SHAP analysis results. Red dots represent higher feature values and blue dots indicate lower ones, while the horizontal distribution of SHAP values reflects both the direction and strength of each feature’s impact on the model prediction. As shown in Fig. 14, fertility-sparing surgery and omentectomy have particularly strong influences on recurrence risk prediction. Through the SHAP analysis results, it could be known that the importance of each clinical feature in recurrence prediction and further improve the interpretability of the model.

## Discussion

BOTs account for 10%−20% of ovarian epithelial malignant tumors [15]. They predominantly affect women of childbearing age, with a mean age at diagnosis of 46 years, approximately 10–20 years younger than patients with invasive ovarian epithelial tumors. Due to their frequent detection at an early stage, BOT patients typically exhibit favorable survival outcomes, with 5-year survival rates exceeding 96% [16] . Despite this excellent prognosis, BOTs are associated with substantial recurrence rates, which represent a key clinical challenge. Our study included 660 BOT patients followed for 12–173 months. Recurrence occurred in 76 patients (11.52%), consistent with previous reports indicating BOT recurrence rates of 5%−20%^6,7^.

The original dataset comprised 88.59% non-recurrent cases and 11.41% recurrent cases, indicating a significant class imbalance. Such imbalance can negatively impact model predictive performance. To mitigate this issue, researchers have proposed solutions at both the data and algorithm levels. Data-level approaches primarily involve resampling techniques to adjust the class distribution toward balance. Common resampling methods include oversampling, undersampling, and hybrid approaches. Oversampling increases the number of minority class instances to approximate the majority class, thereby achieving balance. The SMOTE algorithm, a widely used oversampling method, reduces the overfitting risks associated with traditional oversampling. Studies have demonstrated SMOTE’s effectiveness in significantly enhancing model performance [17, 18]. Specifically, Afrin et al. showed that SMOTE-processed balanced datasets yield significantly better-performing prediction models in regression analysis and random forests compared to imbalanced data [17]. Similarly, Ramezankhani et al. found that SMOTE-enhanced balanced datasets improved performance across neural networks, Naive Bayes, and decision trees for diabetes risk prediction [18].

The Cox proportional hazards regression model remains the most widely used traditional method in survival analysis. However, it requires strict adherence to two key assumptions: proportional hazards and log-linear relationships between variables. This model also faces challenges in capturing interaction effects among multidimensional clinical features. In the era of large-scale medical data, comprehensive utilization of patient characteristics for prognostic prediction has become crucial. However, complex nonlinear relationships among these features often violate the Cox model’s proportional hazards and log-linear assumptions. While previous studies have developed nomograms for BOT recurrence prediction [19], three key limitations persist when using Cox-based nomograms: First, the Cox foundation limits the handling of complex nonlinear relationships in the data. Second, it processes large datasets inefficiently and fails to capture variable nonlinearities. Third, its assumption of constant hazard ratios between patient groups contradicts clinical reality where mortality risks evolve differently across stages/pathologies over time. Significant variations in survival trends exist even among patients at the same stage. This complexity demands more sophisticated analytical approaches. Furthermore, inherent Cox model limitations constrain the performance ceiling of nomograms, with large datasets offering limited opportunities for performance enhancement. We therefore developed a neural network model with fewer data restrictions, a flexible architecture, and superior nonlinear modeling capabilities. This approach overcomes traditional limitations and shows promise as a future standard for survival analysis.

The neural network-based multi-task logistic regression model developed in this study offers three key advantages: First, it effectively captures nonlinear relationships. Unlike traditional models, deep neural networks can identify complex nonlinear patterns between exposure variables and clinical endpoints through multiple layers of activation functions. This capability is particularly valuable when handling incomplete follow-up data. Second, the model demonstrates superior multi-source data integration capacity. The neural network architecture automatically extracts meaningful features from diverse data types (clinical, hematological, radiological, and genetic) without requiring extensive preprocessing, enabling comprehensive analysis of multifaceted oncology data. Third, the model exhibits dynamic performance scalability. The deep learning framework resists performance plateauing and continuously enhances predictive accuracy as data volume increases, thereby effectively surmounting the inherent data saturation constraints of traditional modeling approaches.

In this study, multivariate Cox regression analysis identified seven independent risk factors for BOT recurrence: premenopausal status, laparoscopic surgery, tumor capsule rupture, advanced clinical stage, no lymph node dissection, bilateral tumors, and FSS. In comparison, the N-MTLR model identified nine predictors: FSS, comprehensive staging surgery, comorbidities, FIGO stage, age, omentectomy, lymphadenectomy, parity, menopausal status, and peritoneal implants. Given that BOTs predominantly affect young women, many of whom have fertility requirements, surgical approach selection can impact both reproductive potential and disease prognosis—findings reflected in these risk factors. For clinical implementation, the model can be integrated into decision-support systems where clinicians input these variables to automatically generate recurrence risk assessments, enabling identification of high-risk patients who may benefit from tailored management strategies, including: (1) definitive surgery after childbearing completion [20] or (2) intensified surveillance protocols [21], depending on individual circumstances. The recurrence rate was higher among patients who underwent FSS, who were typically young nulliparous women. The performance of comprehensive staging procedures, including omentectomy and lymphadenectomy, was closely associated with BOT recurrence. Some patients with comorbidities may avoid extensive surgery, which could be related to recurrence. FIGO stage was strongly associated with recurrence, particularly the presence of peritoneal implants, with higher stages correlating with greater recurrence probability. These nine factors demonstrated significant associations with BOT recurrence, with feature importance analysis via SHAP identifying FSS and omentectomy as the most influential predictors. This enhances both model interpretability and clinical utility, while aligning with established surgical risk factors for BOT recurrence, thereby validating the model’s biological plausibility.

The N-MTLR model outperformed the traditional Cox regression model on the internal validation set in terms of specificity, PPV, NPV, accuracy, and AUC. These findings indicate that the proposed model can effectively predict the risk of postoperative recurrence in patients with BOTs. The development of the N-MTLR model facilitates the accurate, sensitive, and specific prediction of BOT recurrence, particularly in the current era of evidence-based and personalized medicine. For patients identified as high-risk by the N-MTLR model, targeted preventive measures and appropriate surgical interventions can be implemented to delay BOT recurrence, especially among those with fertility requirements. For high-risk patients predicted by the N-MTLR model: Patients without adequate follow-up opportunities or patients who have undergone FSS and completed their reproductive plans. Radical surgery is recommended after successful delivery to reduce recurrence risk. Patients with the ability to maintain regular follow-up can be managed with long-term surveillance. Furthermore, the variables used in the proposed model can be obtained through simple medical history and routine clinical tests, avoiding complex procedures or invasive and expensive examinations, and without compromising patient privacy. Thus, the model demonstrates potential for wide clinical application.

Model validation is a critical step in ensuring prediction accuracy and clinical reliability. To rigorously evaluate our N-MTLR-based BOT recurrence prediction model, we implemented comprehensive external validation—the gold standard for assessing model generalizability. External validation examines model performance in independent patient cohorts, establishing both the reproducibility and clinical translatability of predictive algorithms. We employed two distinct validation strategies: (1) Temporal validation using chronological cohorts from our institution demonstrated sustained predictive accuracy, with AUC values maintaining > 0.75 at 2-, 4-, and 7-year postoperative intervals. The model achieved accuracy > 0.70, PPV > 0.65 and NPV > 0.65, confirming temporal robustness. (2) Spatial external validation at the Second Affiliated Hospital of Jilin University yielded AUC values consistently > 0.70 across all timepoints, with particularly strong performance in accuracy > 0.65, sensitivity > 0.80, and NPV > 0.96. These metrics confirmed the model’s spatial generalizability. These validation results provide compelling evidence for the model’s: (i) temporal stability across different treatment eras; (ii) institutional transferability; (iii) potential for clinical implementation; and (iv) superior predictive performance compared to existing tools. The maintained accuracy across both validation paradigms indicates robust feature selection and appropriate model architecture, supporting its immediate clinical adoption with the expectation of reliable performance.

However, the present study has several limitations that should be acknowledged. First, given that the mean time to recurrence of BOTs is 5 years or longer and that BOTs constitute a relatively small proportion of ovarian tumor surgeries at our center, future prospective studies are warranted to perform temporal external validation on our institutional cohort. Second, in this study, the follow-up duration information as a static input variable to make the proposed model as a pseudo-generates time-dependent probability output model, enabling estimation of recurrence risk variation over time. In future work, we plan to extend our current N-MTLR model to achieve temporal dynamics representation of time-dependent probability outputs. That is to say, we will explore generating time-to-event risk trajectories to enhance intuitive visualization of individual risk evolution across follow-up duration. With the additional contents and future developments, we believe the clinical interpretability and academic value of our study will be further strengthened. Third, significant controversies remain regarding the optimal surgical extent, selection of surgical approach, applicability of FSS, choice of postoperative adjuvant therapy, and follow-up protocols for BOTs. Fourth, multicenter trials employing standardized protocols are needed to ensure consistency in diagnostic and therapeutic practices across institutions. Finally, international validation involving diverse populations would further enhance the generalizability and external validity of the model.

## Conclusion

In this study, the SMOTE was employed to address class imbalance in the original dataset. We developed a neural network-based multi-task logistic regression model for predicting BOT recurrence. The model demonstrated robust nonlinear fitting capabilities and exhibited excellent predictive performance for assessing postoperative BOT recurrence risk. This innovative approach can provide more accurate risk stratification and individualized prognostic evaluation in clinical practice, thereby facilitating the optimization of treatment decisions.

## Data Availability

No datasets were generated or analysed during the current study.
